# Sixth Nerve Palsy From Multiple Myeloma in Central Nervous System: Case Series and Review of Current Literature

**DOI:** 10.7759/cureus.40998

**Published:** 2023-06-26

**Authors:** Nishanth Thalambedu, Prashanth Damalcheruvu, Yetunde Ogunsesan, Tanvi Patel, Ramya Bachu, Trilok Shrivastava, Munawwar Hussain, Jaskirat Sethi, Sharmilan Thanendrarajan

**Affiliations:** 1 Internal Medicine, University of Arkansas for Medical Sciences, Little Rock, USA; 2 Radiology, University of Arkansas for Medical Sciences, Little Rock, USA; 3 Hematology and Oncology, University of Arkansas for Medical Sciences, Little Rock, USA

**Keywords:** abducens nerve palsy, relapsed refractory multiple myeloma, multiple myeloma cns involvement prognosis, sixth cranial nerve palsy, multiple myeloma and cns involvement

## Abstract

We reported two cases of the central nervous system (CNS) multiple myeloma (MM) with unusual presentation of sixth nerve palsy. The first patient developed in the setting of newly diagnosed MM and the second patient in the relapsed refractory setting. One underwent surgery, and the other received radiation. Both patients received systemic chemotherapy and noted improvement. We also performed a comprehensive literature review of previously published cases of sixth nerve palsy from MM. This review highlights the importance of recognizing this presentation of CNS multiple myeloma to avoid delays in diagnosis and to get appropriate management in time.

## Introduction

Multiple myeloma (MM) involving the central nervous system (CNS) has various manifestations along its spectrum. The most common presentations are bone lesions as a part of systemic disease, causing significant pain. On the contrary, localized plasmacytomas can arise from the cranium (intraosseous) or dura, meninges or parenchyma (extramedullary) with or without evidence of systemic disease [1].

The clinical symptoms of CNS plasmacytomas depend on the part involved ranging from extremity weakness, change in mental status, gait and visual disturbances to cranial nerve palsies [2]. The diagnosis will be challenging especially when presented with isolated cranial nerve palsies due to multitudinous pathologies mimicking the presentation [3]. It is important to recognize this presentation of MM to avoid delay in diagnosis and to hasten management. Here, we present two cases of CNS MM manifested as sixth nerve palsy. We also reviewed the literature of previously reported cases of sixth nerve palsy from MM.

## Case presentation

Case 1

**Figure 1:**
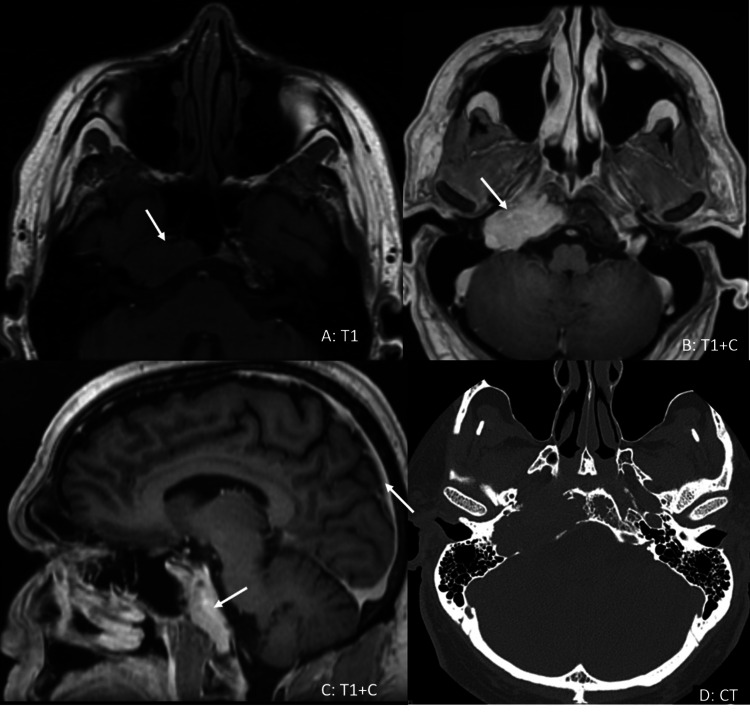
MRI findings Axial pre-contrast T1 (A) and post-contrast T1-weighted images (B) demonstrate a large expansile mass (arrow) involving the right side of the clivus, petrous apex and right jugular foramen. Sagittal post-contrast T1-weighted images (C) demonstrate an enhancing mass replacing the entire right side of the clivus. Axial bone window CT images (D) show an expansile lytic lesion (arrows) on the right side of the clivus and petrous apex, extending to the jugular foramen. The mass involves the expected location of Dorello’s canal.

**Figure 2:**
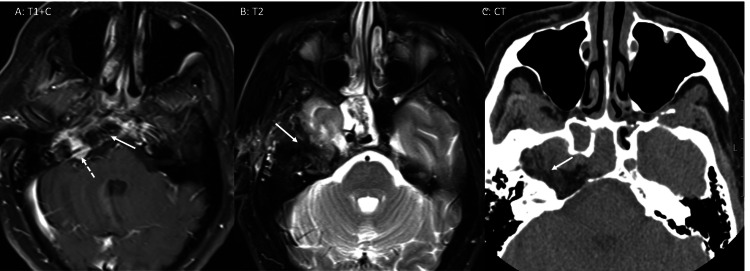
Follow-up MRI after resection of the mass Follow-up MRI after resection of the mass shows post-operative changes due to the presence of a fat graft (arrows in A, B, and C). A small residual enhancing lesion is present (dashed arrow in A) in the jugular foramen.

A 55-year-old Caucasian male presented with a one-month duration of double vision mainly worse on looking to the right side, numbness of the right lateral aspect of lips and gums, right-sided hearing loss and tinnitus. He was also evaluated by an otolaryngologist and found to have asymmetric sensorineural hearing loss (SNHL) on the audiogram. He was evaluated by an ophthalmologist and diagnosed with right sixth nerve palsy. Computerized tomography (CT) scan showed a 4.2 cm × 2 cm expansile mass in the right jugular foramen with erosive changes of the adjacent bones including the right clivus, occipital condyle, petrous apex, erosion of the carotid canal and posterior wall of the sphenoid sinus with the extension of the lesion into the sphenoid sinus. There was no evidence of extension to the middle ear or involvement of the inner ear structures. Magnetic resonance imaging (MRI) confirmed the same findings as CT (Figures 1A-1D). He underwent an uneventful craniotomy with the excision of a skull tumor. His double vision improved significantly post-surgery. His post-surgical MRI revealed a small residual enhancing lesion in the jugular foramen (Figures 2A-2C). A biopsy of the tumor showed plasmacytoma.

On further workup, bone marrow biopsy showed 6% plasma cells on aspirate and 15%-20% on core biopsy. The minimal residual disease (MRD) test was positive at 2.83%, and flow cytometry showed CD138, CD56, and CD38 positive and negative for CD45, 19, and 20. Fluorescent in situ hybridization (FISH) multiple myeloma panel showed +1q21.

Lab work showed a hemoglobin level of 12.6 g/dl, calcium 10.1 mg/dl, serum creatinine 1.1 mg/dl, M component in serum 0, Bence-Jones proteinuria at 272 mg in 24 hours urine with a free kappa light chain in the urine immunofixation, with kappa light chain level of 102.8 mg/dL, with a kappa lambda ratio of 121.8, with decreased IgA and IgG levels and normal IgM levels. He was found to have several bone lesions defined by the positron emission tomography (PET) scan and the MRI studies including the sternum, the right shoulder, the scapula, the left clavicle and the skull base with very likely extramedullary disease in the right pelvic soft tissue measuring up to 5 cm in size. He was diagnosed with kappa light chain multiple myeloma, and he received induction chemotherapy with daratumumab and carfilzomib, dexamethasone, thalidomide, cisplatin, adriamycin, cyclophosphamide and etoposide (KDT-PACE). His ocular symptoms have completely resolved, and he will be undergoing an autologous stem cell transplant (ASCT) shortly.

Case 2

**Figure 3:**
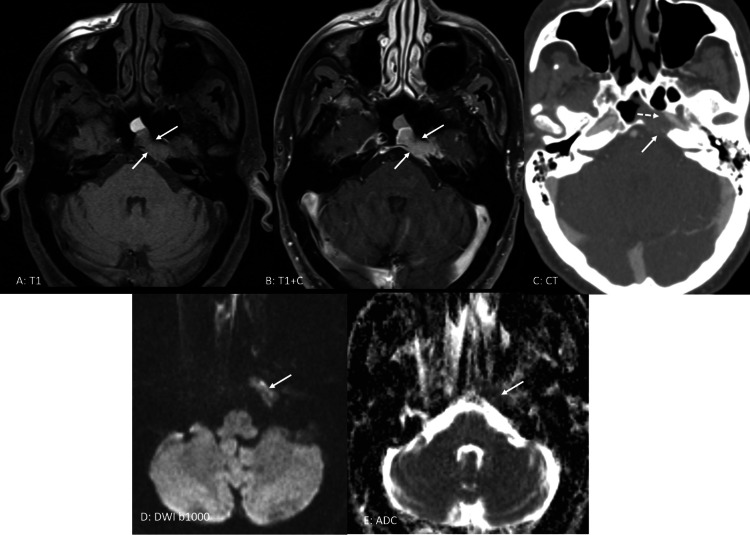
Images of skull base Pre-contrast (A) and post-contrast (B) axial T1-weighted images of the skull base demonstrate a mildly expansile, homogeneously enhancing mass (solid arrows) in the left side of the clivus in the expected location of the Dorello’s canal, the osteofibrous canal in the petrous apex containing the abducens nerve. Axial contrast-enhanced CT image (C) demonstrates an enhancing mass causing lytic destruction of bone in the clivus and petrous apex (solid arrow) with the erosion of the carotid canal (dashed arrow). Diffusion-weighted image (DWI) (D and E) demonstrates low signal within the lesion on apparent diffusion coefficient (ADC) map images (arrows) representing restricted diffusion.

A 69-year-old Caucasian male with a past medical history of long-standing multiple myeloma presented with double vision of two weeks duration. He was off of any therapy for his MM when he developed diplopia. Prior to that he was having MM for almost six years and was extensively treated with induction, tandem ASCT and consolidation therapies and received multiple lines of maintenance regimens. On exam, he was noted to have left sixth cranial nerve palsy with no other neurological findings. MRI of his brain revealed a 2.2 × 1.0 × 1.8 cm enhancing mass in the left petrous apex and posterior cavernous sinus. PET scan showed fluorodeoxyglucose (FDG) uptake in the same lesion with no other new lesions or extramedullary disease (EMD) (Figures 3A-3E). His serum myeloma markers remained stable compared to the markers a month prior. He also underwent bone marrow biopsy which was MRD positive but was MRD negative six months ago.

**Figure 4:**
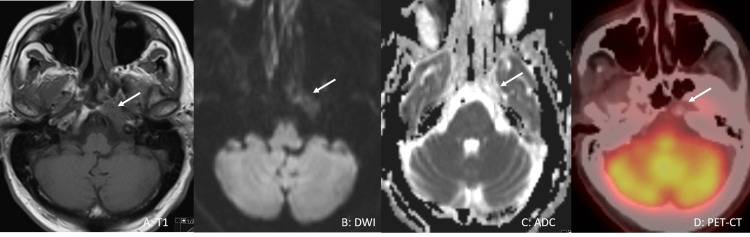
Follow-up MRI Follow-up MRI demonstrates a reduction in the size of the lesion in the left clivus (arrow in A) as well as resolution of diffusion restriction. The lesion now demonstrates a high signal on both diffusion-weighted image (DWI) and apparent diffusion coefficient (ADC) images (arrow in B and C) consistent with facilitated diffusion. Positron emission tomography CT (PET-CT) (D) shows no evidence of fluorodeoxyglucose (FDG) uptake within the lytic lesion (arrow).

Based on the above findings, he was started on high-dose steroids, dexamethasone 40 mg daily for four days. He also underwent radiation to the lesion for five weeks. Post-radiation, his symptoms completely resolved. Imaging also demonstrated a reduction in left clivus lesion size and resolution of diffusion restriction (Figures 4A-4D). Following that he was started on Blenrep 2.5 mg/kg every four weeks. He received a total of three doses, and he was doing well.

## Discussion

We reported two cases of multiple myeloma associated with sixth cranial nerve palsy. Case 1 developed in the setting of newly diagnosed MM, while case 2 developed in the relapsed refractory background. Case 1 was noted to have multiple cranial nerve palsies, but case 2 was noted to have isolated sixth nerve palsy. Both the patients' MRIs clearly showed the origin of the lesion. Case 1 underwent surgery for tumor removal, but case 2 received radiation. Diplopia is resolved in both cases post-operatively and post-radiotherapy, respectively. Both patients received systemic chemotherapy and are currently in remission with no recurrence of neurological symptoms.

Intracranial plasmacytomas are rare plasma cell neoplasms constituting less than 1% of all intracranial tumors and can occur as part of the manifestation of MM or as a solitary (primary) lesion arising from the skull, meninges and the brain [4]. Plasmacytoma of the skull base arises from the clivus or the petrous part of the temporal bone with the body of the sphenoid and the apex of the petrous bones being the most common sites [5]. Because of its close proximity to the cranial nerves, especially the abducens nerve making it the most frequent cranial nerve affected [6]. Every so often, the myeloma lesion extends to adjacent structures and compresses them, leading to additional cranial nerve palsies and neurological symptoms [7].

We performed a PubMed/Embase/Web of Science-based comprehensive literature review for previously published cases of sixth cranial nerve palsy among MM patients until November 2022. The search terms included abducens nerve" OR "sixth cranial nerve" OR "sixth nerve" OR "6th nerve" OR "6th cranial nerve" (Topic) and palsy OR palsies OR paralysis (Topic) and "multiple myeloma" OR "plasma cell myeloma*" (Topic). We reviewed all results and found the following previous publications as outlined below in Table 1.

**Table 1:** Previously published cases of sixth cranial nerve palsy among multiple myeloma patients Previously published cases [8-28]. RR: relapsed refractory; IT Mtx: intrathecal methotrexate; WBRT: whole brain radiation therapy; Rd: Revlimid, dexamethasone; CyBD: cyclophosphamide, bortezomib, dexamethasone; Vd: Velcade, dexamethasone; R: radiation; DV: Daratumumab, Velcade; Post-op: post-operative.

Author	Isotype	Age/Sex	New/RR	Duration of Symptoms	Imaging	Treatment (Chemo vs Radiation)	Time to Resolution of Symptoms
Fitzgerald et al. [8]	IgG	71/f	RR		Leptomeningeal infiltration of right medial petrous apex involving cavernous sinus	IT Mtx + WBRT + Rd	Deceased in 5 weeks
Kaufman et al. [9]	IgG K	44/m	RR	3 weeks	Large skull base lesion in left petrous apex extending into clivus	DV + R	7 months
Badr-El-Dine et al. [10]	Lambda	52/m	New	3 months	Right petrous bone lesion	Right petrosectomy	
Ibekwe et al. [11]	Lambda	53/f	New	6 days	Right clivus lesion	CyBD + R	3 months
Zaino et al. [12]	Kappa	78/m	RR	1 day	Right clivus lesion	Vd + R	4 months
Thiruvengadam and Prayson [13]	Kappa	63/f	New	2 weeks	Right petrous apex bone lesion	Rd	
Kalwani et al. [14]	Lambda	69/m	New	1 week	Clivus lesion	Surgery + R	Post-op
Grisold et al. [15]	Lambda	66/m	RR		Normal	IT + systemic chemo	4 weeks
Cetin et al. [16]	IgG K	50/m	RR		Clivus	Radiation	4 days
Jiang et al. [17]	Lambda	34/f	New	25 days	Sellar mass	Radiation + chemo	
Khalatbari et al. [18]		46/m	New	2 days	Left petrous apex	Surgery + R	4 months
Kashyap et al. [19]	Kappa	55/m	New		Clivus lesion	Chemo + R	Never resolved
Na et al. [20]	IgA Kappa	63/f	New	2 weeks	Left petrous apex		
Yamaguchi et al. [21]	IgA lambda	65/f	New	2 months	Clivus lesion	R + Chemo + Surgery	Post-op
Nakaya et al. [22]	IgA lambda	71/m	New		Sphenoid sinus	Chemo + R	
Reddy et al. [23]	Kappa	52/m	New	6 months	Clivus mass	Chemo + R	
Rai and Patel [24]		68/f	New	2 months	Clivus mass	Radiation	
Duran et al. [25]	Kappa	66/f	New		Clivus mass	Surgery + Chemo + Radiation	Post-radiation
Cortez et al. [26]	IgG Kappa	62/m	New	3 months	Lesser wing of sphenoid	Surgery + Chemo	
Krawczyk et al. [27]		49/m	New		Right cavernous sinus	Chemo + R	Resolved
	IgA	64/f	RR		Left cavernous sinus	Chemo + R	Deceased
		69/m	New		Skull base	Radiation	Deceased
	IgG	70/f	RR		Cavernous sinus lesion	Chemo + R	Deceased
Murthy et al. [28]	Light chain	44/f	New	3 months	Cavernous sinus lesion	Chemo	4 weeks

The sixth nerve palsy in intracranial plasmacytomas can occur in both newly diagnosed and RR backgrounds. The symptoms' duration varies from weeks to months prior to the final diagnosis. The delay in diagnosis might be due to the challenges faced especially in newly diagnosed settings due to the rarity of occurrence with a low index of suspicion and the multitude of pathologies that can cause similar presentations. From Table 1, we noted a majority of abducens nerve palsy among newly diagnosed RR patients with the duration of symptoms ranging from days to months strengthening to maintain a high index of suspicion when evaluating patients with cranial nerve palsies.

Out of many risk factors associated with the development of CNS MM, one study noted that chromosome 17p 13.1 deletion was associated with 89% of CNS MM patients which was not seen in both of our patients [29]. However, case 1 FISH from bone marrow biopsy revealed a gain of 1q21 in a small percentage of nuclei (4.6%); its role in CNS MM is still unclear.

Treatment of CNS plasmacytomas can be approached through three modalities: surgery, radiation and chemotherapy. Surgery is usually reserved for tumors causing significant mass effect. Sometimes it serves as a modality in reaching out for a diagnosis of plasmacytoma [3]. But once the diagnosis of CNS plasmacytoma is made, localized radiation at the recommended dose of 40 Gy in 20 fractions for a tumor less than 5 cm in size and up to 50 Gy for lesions more than 5 cm in size with at least a 2 cm margin surrounding the primary tumor for four to five weeks [30]. Systemic chemotherapy usually follows radiation. The choice of chemotherapy for CNS MM varied across the literature, but the most commonly favored agents include bendamustine, bortezomib, thalidomide, lenalidomide and other immunomodulatory drugs (IMiDs) [8]. From our review in Table 1, almost all patients received radiation with very few exceptions. Both of our patients received radiation followed by systemic chemotherapy, but case 1 underwent surgery for tumor debulking.

## Conclusions

To conclude, we presented two cases of unusual presentations of CNS MM as sixth nerve palsy. The lesion was localized in MRI in both patients. One patient underwent surgery followed by systemic chemotherapy, and another patient underwent radiation first followed by systemic chemotherapy. Both showed improvement in their symptoms. This case series highlights the CNS as an unusual site for MM to start or relapse. A high index of suspicion needs to be maintained. Treatment should be initiated promptly with surgery or radiation followed by systemic chemotherapy.
